# Classifying Motion Intention of Step Length and Synchronous Walking Speed by Functional Near-Infrared Spectroscopy

**DOI:** 10.34133/2021/9821787

**Published:** 2021-04-22

**Authors:** Yufei Zhu, Chunguang Li, Hedian Jin, Lining Sun

**Affiliations:** Key Laboratory of Robotics and System of Jiangsu Province, School of Mechanical and Electric Engineering, Soochow University, China

## Abstract

In some patients who have suffered an amputation or spinal cord injury, walking ability may be degraded or deteriorated. Helping these patients walk independently on their own initiative is of great significance. This paper proposes a method to identify subjects' motion intention under different levels of step length and synchronous walking speed by using functional near-infrared spectroscopy technology. Thirty-one healthy subjects were recruited to walk under six given sets of gait parameters (small step with low/midspeed, midstep with low/mid/high speed, and large step with midspeed). The channels were subdivided into more regions. More frequency bands (6 subbands on average in the range of 0-0.18 Hz) were decomposed by applying the wavelet packet method. Further, a genetic algorithm and a library for support vector machine algorithm were applied for selecting typical feature vectors, which were represented by important regions with partial important channels mentioned above. The walking speed recognition rate was 71.21% in different step length states, and the step length recognition rate was 71.21% in different walking speed states. This study explores the method of identifying motion intention in two-dimensional multivariate states. It lays the foundation for controlling walking-assistance equipment adaptively based on cerebral hemoglobin information.

## 1. Introduction

More than 15 million people per year suffer a stroke, and this is the second-greatest cause of death across the world [1]. The muscle weakness, spasticity, impaired sensory-motor control, and loss of cognitive function that can occur following a stroke mean that patients have a raised possibility of falling over and suffering significant energy loss during normal walking [2, 3]. Moreover, the aging population has become a global issue, with approximately 21% of the world's population forecast to be aged 60 or above by 2050 [4]. The attendant problem is the significant motor dysfunction that may result from the decline in cognitive function [4]. Furthermore, the number of accident victims suffering from spinal cord injury (SCI) and severely injured lower limbs continues to increase. For these patients, travel has become a major challenge, and reliance on a wheelchair may result in a lack of exercise and reduced active motion intention. This can lead to an accelerated decline in brain functions with age [5–7]. Therefore, there is strong social significance in increasing the intelligence of walking-assistance equipment so as to help these patients walk independently using their motion intention.

Recently, studies based on electromyography (EMG) signals [8, 9] and biomechanical sensors [10, 11] have made great progress in controlling walking-assistive exoskeletons through the subjects' motion intention. However, patients with restricted physical activity are not conducive to the optimal judgment of EMG signals or biomechanical sensors. Therefore, brain signals have become the preferred method of assisting patients to walk through the determination of their motion intention.

Research on brain-computer interfaces (BCIs) has made tremendous progress recently, particularly for electroencephalography (EEG) technology [12–16]. Severens et al. [13] confirmed that brain signals related to walking could be classified rapidly and reliably, while Zhang et al. [15] determined the cortical plasticity triggered by the brain-computer interface through longitudinal experiments. The ability to control external devices based on the use of neuroimaging techniques will be extremely helpful to patients with motor dysfunction. However, the EEG test environment should take into account the influence of the local electromagnetic environment. And large-scale body movements, such as head movements, can also cause obvious interference to the EEG. In this manuscript, the subject of the test is a walking experiment, which is often accompanied by more and larger limb movements, and the test environment is nonexperimental. These factors restrict the application of EEG technology.

Functional near-infrared spectroscopy (fNIRS) supports continuous testing in the natural environment. The excellent usability and reduced sensitivity to head motion artifacts mean that fNIRS-BCI may be able to assist in the movement of lower limbs [17, 18]. Some fNIRS studies have identified the activation regions of the brain during walking [19–21]. For example, Kim et al. and Mihara et al. found that the activation of the sensorimotor cortex (SMC), premotor cortex (PMC), and supplementary motor area (SMA) is largely associated with an increase in walking speed [19, 20], and the main activation area due to changes in the step length is believed to be the prefrontal cortex (PFC) [21]. Simultaneously, research on the classification of multiple gait motion states has also made strong progress [22–24]. These studies have focused on step length and walking speed, but there is little research on the simultaneous recognition of these two parameters.

In this study, a method is developed to identify subjects' spontaneous motion intention under different levels of step length and synchronous walking speed. Regarding the method of identifying subjects' motion intention, all walking states tested in this study were simultaneously identified, to determine a precise motivation quickly and accurately. We believe that this study lays the foundation for the control of walking-assistance equipment based on patients' cerebral hemoglobin information, thus helping patients to restore their ability to walk independently.

## 2. Experiment

### 2.1. Subjects and Instrument

Thirty-one healthy subjects (twenty-three male and eight female, 22 ± 4 (mean ± s.d.) years old) were recruited by the School of Mechanical and Electronic Engineering, Soochow University. All subjects were right-handed and had no neurological abnormalities. Moreover, all subjects gave their written informed consent before the experiments, and it was the first time they had participated in such tests. The subjects were instructed to walk under six given sets of gait states (small step (SP) with low/midspeed (LD/MD), midstep (MP) with low/mid/high speed (LD/MD/HD), and large step (LP) with midspeed (MD)). During the walking experiment, the subjects' total hemoglobin (totalHb), oxygenated hemoglobin (oxyHb), and deoxygenated hemoglobin (deoxyHb) levels were measured by a FORIE-3000 optical topography system [22]. This system consists of eight emitters and eight detectors and measures wavelengths of 780 nm, 805 nm, and 830 nm. The sampling period was set to 0.13 s.

### 2.2. Cortical Regions and Paradigm

**Figure 1:**
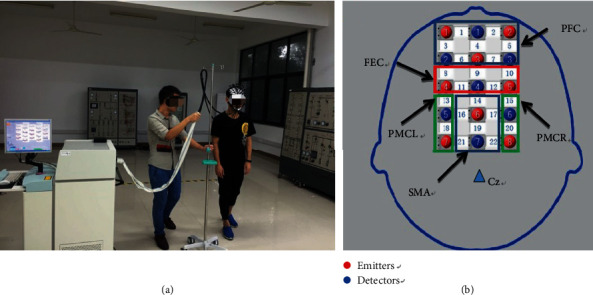
(a) The experiment diagram: the subject was walking in the state of SL, the right one was the subject, and the left one was a researcher walking with the subject carrying the weight of the fNIRS cables. (b) The arrangement of optodes: the channel 1 to channel 7 were approximately in the PFC area, the channel 8 to channel 12 were approximately in the FEC area, the channel 13 and channel 18 were approximately in the PMCL area, the channel 15 to channel 20 were approximately in the PMCR area, and the channels 14, 16, 17, 19, 21, and 22 were approximately in the FEC area.

Based on the research of Kim et al., Mihara et al., and Holtzer et al. [19–21], the international 10-20 system [25, 26] and the Brodmann partition map [21], the PFC, frontal eye cortex (FEC), SMA, and PMC were tested (Figure 1). The distance between each detector and emitter was fixed at 3 cm. The Cz point is the intersection of the left to right earlobe and nasion root to occipital tuberosity; the distance between the Cz point and the detector 7 was 3 cm.

**Figure 2:**
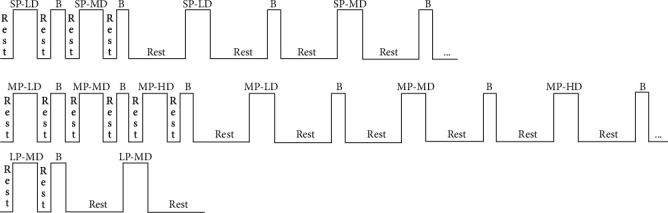
The specific process of the whole experiment. The vertically written “Rest” represents that the rest time is approximately 10 s; another writing represents that the rest time is approximately 40 s. “B” represents the recession process; the subject must return to the starting point after completing a movement. SP-LD: the gait of small step with low speed; SP-MD: the gait of small step with midspeed; MP-LD: the gait of midstep with low speed; MP-MD: the gait of midstep with midspeed; MP-HD: the gait of midstep with high speed; LP-MD: the gait of large step with midspeed.

According to the feedback from a medical doctor, all subjects were required to walk with six gait states during the period of rehabilitation training. And because of the limitation of the transmission lines' length, the length of the road was approximately 4.4 m (Figure 1). For the weight of the fNIRS cables, a researcher would walk with subjects carrying it. To reduce any initial tension or discomfort felt by the subjects, they were asked to repeat each of the six gait states twice. Through the experiment, it was found that a long period of the test would irritate the scalp. Thus, during the first set of gait tasks, the subjects were asked to take a break of approximately 10 s between each of the two walking tasks, whereas the rest interval was increased to approximately 40 s during the second set. The first set of gait was used to adapt to the experiment. The scheme of the experimental protocol is shown in Figure 2; all movements follow this scheme. Sixteen subjects started walking with their right foot, and fifteen started walking with their left foot.

The starting point and ending point were marked previously, but the moment of the start of each movement was controlled spontaneously by the subjects. For the step length, the small step (0.45 m) was about nine steps in this fixed distance, the midstep (0.75 m) was about six steps, and the large step (1.1 m) was about four steps. Walking speed was also controlled by the subjects based on their normal walking speed. The low speed (0.2 m/s) must be slower than normal speed (0.5 m/s), and the fast speed (0.8 m/s) must be faster than normal speed. During the training time, the researcher will time each state, to make sure that each gait parameter was different. Before the experiments, all subjects were asked to wash their hair and ensure their scalp was dry. Subjects were informed of the experimental procedure as well as certain details that should be adhered to during the experiment, specifically, that subjects cannot count during the rest and task, the head cannot swing significantly, and the arms should remain in a natural state.

### 2.3. Data Analysis

The six gait patterns were classified under the different levels of step length and walking speed (Table 1). That is, under the different step lengths, states SP-LD and MP-LD were considered as the low speed state and so on. These new states were identified based on the totalHb measurement and the difference between oxyHb and deoxyHb. All calculations and analyses were conducted in MATLAB R2016a, and only the data obtained from the second test were used for analysis.

As the acquired brain signals exhibited some zero-drift components, this zero drift is known to affect the results of power spectrum analysis and wavelet packet decomposition [27, 28] and is not conducive to the determination of effective feature vectors. In this study, the 180 points before movement were selected for zero-drift processing using the mathematical morphology filter [29]. This data length was selected to meet the principle of wavelet packet decomposition. Furthermore, to reduce the differences between individuals, the data were then normalized according to

(1) $$\begin{array}{c} \text{xN}=2*\left[\frac{x-\min}{\max-\min}\right]-1, \end{array}$$

where *x* denotes the time series data from one channel with the zero drift removed; *x*_max_ and *x*_min_ denote the maximum and minimum values of the same time series data, respectively; and xN represents the normalized time series data.

To determine the main frequency range and band interval, power spectrum analysis was performed using a rectangular window. The continuous frequency maps of all channels in each state were studied, and the main frequency range was determined by observing the active frequency range of each channel. The band interval was confirmed based on the intervals of the primary and secondary frequency peaks.

According to the sampling period of hemoglobin signals and the power spectrum analysis results, the main frequency band and band interval give the band interval of the wavelet packet decomposition. The normalized data were decomposed using wavelet packets, and time series were reconstructed for important subbands. Then, as for each subband, the rate of change of all channels was used to extract feature vectors. As the aim of this study was to identify the motion intention of different states, the average rate of eight points (approximately one second) before the actual movement was used for the final analysis to identify the motion intention in real time.

Twenty subjects were selected as the training set (15 males and 5 females; 10 subjects started walking with the right foot and 10 started with the left). Under the different step lengths and walking speeds, the six walking states were divided into three new states for each dimension (step length or walking speed). As an example of the feature selection, consider the low speed state containing the SP-LD and MP-LD states (Table 1).

Wavelet packet decomposition not only decomposes the low-frequency part but also decomposes the high-frequency part. The frequency band is divided into different levels, the corresponding frequency band is selected adaptively to analyze the signal characteristics, and the corresponding signal spectrum improves the capability of time-frequency analysis. After wavelet packet decomposition, each state admits a 6 × 22 matrix (6 frequency bands × 22 channels). For the small step state, the average matrix M1 (where M denotes a matrix) of SP-LD and SP-MD was calculated. The absolute values of the coefficient of variation (CV), which was used to find out the important channel with the same characteristics, were also calculated based on each element of the SP-LD and SP-MD matrices, and these values formed a second matrix, M2. To determine the main feature element (channels in different frequency bands) without obvious differences in the SP-LD and SP-MD states, the bottom 50% of the CV values were assigned values of 1, whereas the others were assigned values of 0. These values formed a new matrix, M3. To identify the main channels with obvious activation and inhibition in different frequency bands, the top 20% and the bottom 20% of the average matrix were also assigned values of 1, and the other elements were set to 0 (giving M4 and M5, respectively). M3 was then combined with M4 and M5 in turn to give M6 and M7. If one element satisfied the above two conditions simultaneously, the corresponding channel in the corresponding frequency was identified as a key channel. In this way, the obviously activated and inhibited channels could be extracted, and simultaneously, there is no distinct difference between the SP-LD and MP-LD states. This is helpful in determining the common characteristics of the low speed state, even for different step lengths.

To reduce the influence of different head sizes, the 22 channels were subdivided into 22 regions according to a spatial location (Table 2). If a key channel was found in one of the newly demarcated regions, that region was defined as a key region. The number of key channels in each key region was counted, and the results were used to form matrices M8 and M9 (quantized matrices: 6 frequency bands × 22 regions).

To further ensure that the selected features were suitable for most of the 20 subjects, a frequency statistical method was used to count the probability of the key regions and the number of key channels in each key region. If the percentage of the frequency exceeded 60% (12/20), this region was defined as a feature region, and the number of key channels in this region was the number of occurrences. For the other five new states (Table 1), the feature extraction method was as described above.

**Figure 3:**
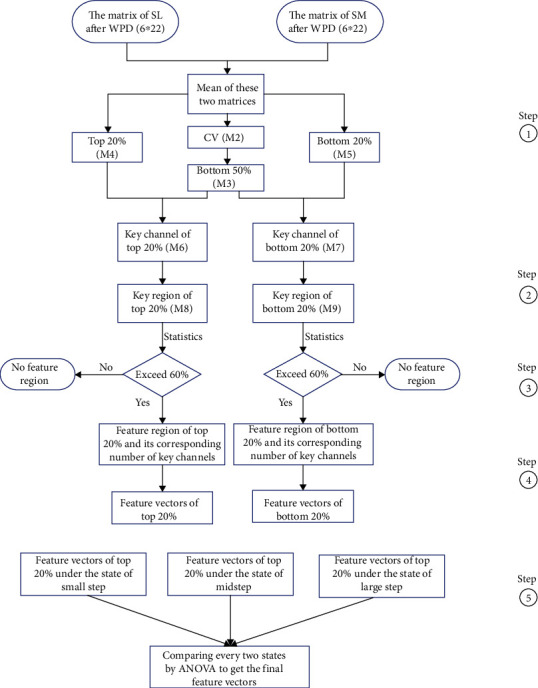
The detailed steps of the calculation flow. WPD: the method of wavelet packet decomposition; SP-LD: the gait of small step with low speed; SP-MD: the gait of small step with midspeed; CV: the coefficient of variation; M1 to M9: different matrices calculated in each step. The illustration on the right side of the figure corresponds to the steps in the text.

According to the feature regions and their corresponding number of key channels, the blood oxygen concentrations in M1, rather than the quantized values, were calculated as the preliminary characteristics for pattern recognition. For example, in one feature region in the top 20%, if the key channel number was 1, the channel with the maximum concentration value in this region was the final feature for this step. Moreover, the entire frequency band was divided into two independent parts (0-0.09 Hz and 0.09-0.18 Hz). If continuous feature regions appeared in one part, these feature vectors were combined to form a new feature vector. The above calculations were performed for each state. The detailed flow chart is as follows Figure 3.

To further identify different feature vectors for the different states, consider the example of the top 20% condition under different walking speeds (Table 1). One-way ANOVA was used to compare every pair of states, and if the corresponding *p* value was less than 0.05, this region was treated as a feature vector for pattern recognition. The same method was applied for the different step lengths.

For the training set, twenty subjects were selected to identify three new states under the different step lengths and walking speeds. For these two conditions, the subjects forming the training set and the testing set were the same. All the feature vectors for the totalHb and the difference between oxyHb and deoxyHb were recombined through the genetic algorithm (GA) [30]. The genetic algorithm simulates population iteration and evolution through chromosome selection, crossover, and mutation, which is very suitable for super parameter optimization of the model. The genetic algorithm consists of the following steps: initialize the group; calculate fitness, chromosome selection, chromosome decoding, crossover, and mutation; calculate fitness and chromosome decoding; and stop searching. The calculation formulas for crossover probability and mutation probability are as follows:

(2) $$\begin{array}{c} p_{c}=\left\{\begin{array}{cc} p_{c1}-\frac{\left(p_{c1}-p_{c2}\right)\left(f^{\prime }-f_{\text{avg}}\right)}{f_{\max}-f_{\text{avg}}}, & f^{\prime }\geq f_{\text{avg}},\\ p_{c1} & f^{\prime }<f_{\text{avg}}, \end{array}\right. \end{array}$$

(3) $$\begin{array}{c} p_{m}=\left\{\begin{array}{cc} p_{m1}-\frac{\left(p_{m1}-p_{m2}\right)\left(f-f_{\text{avg}}\right)}{f_{\max}-f_{\text{avg}}}, & f\geq f_{\text{avg}},\\ p_{m1}, & f<f_{\text{avg}}, \end{array}\right. \end{array}$$

where *f*_max_ represents the largest fitness value in the contemporary population, *f*_avg_ represents the average fitness of the contemporary population, *f*′ represents the larger fitness value of the two individuals to be crossed, and *f* represents the fitness value of the individual to be mutated. Also, *p*_*c*1_, *p*_*c*2_, *p*_*m*1_, and *p*_*m*2_ represent the critical values of crossover and mutation probability, respectively.

It can be used to optimize features and model parameters. Under this feature vector combination, nineteen subjects were selected for training data and one for testing data, a total of twenty combinations according to the different permutations. And the SVM algorithm [31] was used to calculate its recognition accuracy. The final recognition accuracy of this feature vector combination was their mean value. Finally, through the twenty thousand iterations, the highest recognition accuracy and its corresponding feature vector combination were found for the above two conditions, respectively. Then, according to the feature vector combinations of calculation, these twenty subjects were defined as the training set to calculate the final recognition accuracy of the remaining 11 subjects (11 subjects × 6 gait parameters).

## 3. Results

### 3.1. Data Preprocessing

**Figure 4:**
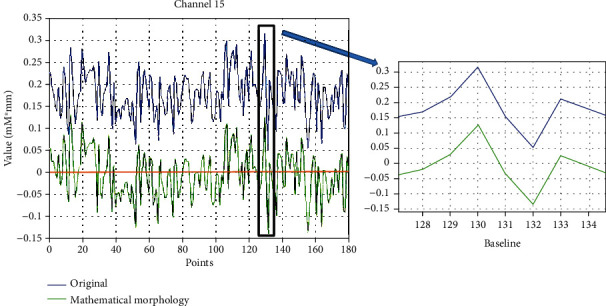
The comparison chart of subject 1's channel 15 under the state of small step and low speed. The results of different channels in other states are similar to this one: the blue line represents the original signal, the green line represents the data after mathematical morphology, and the yellow line is the baseline—its value is zero.

The data with the zero-drift component removed are shown in Figure 4. There was no time delay using this method. After normalization, the range of all values is [-1, 1].

### 3.2. Power Spectrum Analysis

**Figure 5:**
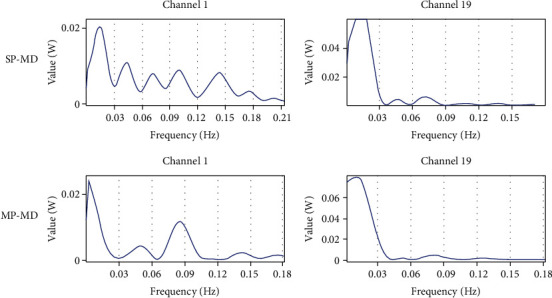
Subject 1's continuous frequency map of channel 1 and channel 19 under the states of small step with midspeed (SP-MD) and midstep with midspeed (MP-MD). The results of different channels in other states are similar to this one.

After normalization, power spectrum analysis was performed on each channel separately. Typical figures are shown in Figure 5. By observing the main frequency range of different channels in different states, it was found that the main power was concentrated from 0 to 0.18 Hz. Therefore, the main frequency band was defined as 0–0.18 Hz. Moreover, the interval between each crest (i.e., the interval between primary and secondary frequencies) was approximately 0.03 Hz. Therefore, the subband interval was defined as 0.03 Hz.

### 3.3. Feature Extraction

Because the sampling period of the hemoglobin signal was set to 0.13 s, the signal sampling frequency was approximately 7.7 Hz. According to the power spectrum analysis results, the data were decomposed into 128 layers by wavelet packet decomposition, and the subband interval of each layer was 0.03 Hz. Next, the slopes of all channels were calculated, and the eight points prior to the actual movement were averaged for the final analysis.

**Figure 6:**
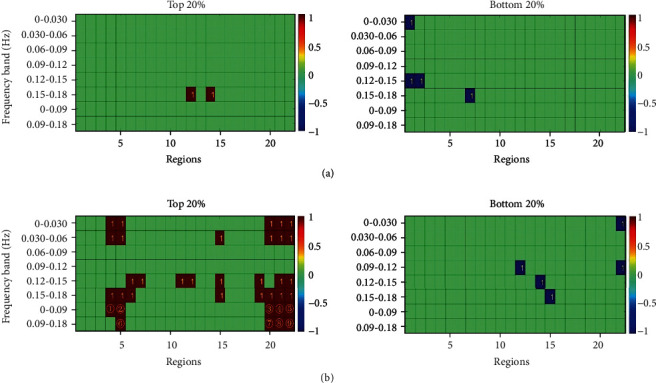
The feature vectors under the different levels of step length: (a) represents the important feature vectors under the total hemoglobin parameter; (b) represents the important feature vectors under the difference between oxygenated hemoglobin and deoxygenated hemoglobin parameters. The red square presents that these feature regions were under the top 20%, and the blue one represents that the feature regions were under the bottom 20%. The digit in the red and blue square represents the key number in this region. For example, if the digit “1” was in the red square, it means that the feature vector is a channel with the largest value in this area; if the digit “1” was in the blue square, it represents the smallest one. The specific representation of serial numbers is shown in Table 3.

**Figure 7:**
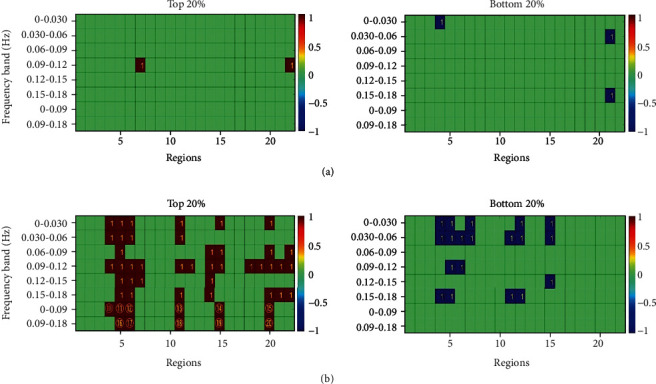
The feature vectors under the different levels of walking speed: (a) represents the feature vectors under the totalHb parameter; (b) represents the feature vectors under the difference between the oxyHb and deoxyHb parameters. The specific representation of serial numbers is shown in Table 3.

According to the method described above for finding the feature vectors, the corresponding feature vectors under the different step lengths and walking speeds were determined. ANOVA is the analysis of test data to verify whether the mean values of multiple normal populations with the same variance are the same, then judge whether the influence of each factor on the test index is significant. After selecting the most appropriate features using the statistical technique and one-way ANOVA, the results displayed in Figures 6 and 7 were obtained. The specific representation of the serial number is shown in Table 3.

For the above feature vectors, the GA and SVM algorithm were used to identify the three new states under the conditions of different step lengths and walking speeds, respectively. Finally, the recognition rates of the testing set were found to be 71.21% (47/66) and 71.21% (47/66), respectively (Table 4).

## 4. Discussion

The main purpose of this study was to develop a method of identifying the motion intention of patients based on their brain signals. The results could be used to control walking-assistance equipment through patients' motion intention and allow them to walk independently. In practical applications of BCI technology, the real-time operation is very important. Hence, in this study, the motion intention prior to the actual movement was selected as the analysis object for identifying the subjects' movements. Moreover, to fully mobilize the subjects' motion intention, the subjects were not given any reminders in the initial stage, so that all movements were spontaneously controlled by the subjects.

This study did not focus on identifying the fixed walking speed or step length. The six gait states, which contain almost all of the walking conditions required by patients, were classified under the conditions of different step lengths and walking speeds. Moreover, we proposed a method for identifying these six gait states at the same time under both conditions, and the recognition rates of walking speed (for different levels of step length) and step length (for different levels of walking speed) were found to be 71.21% and 71.21%, respectively. In detail, under the condition of different step lengths, the proposed method can identify three different walking speeds for a normal gait. Therefore, the experimental process does not limit the subjects' step size. Similarly, for the condition of different walking speeds, the experimental process does not limit the subjects' speed. By using this method, patients may walk according to their motion intentions and without training, when using walking-assistance equipment, rather than walk in a fixed speed level or step size. This can increase adaptability and fully mobilize the patients' initiative to encourage participation.

In the feature extraction stage, our approach is not limited to the whole filtered band [24, 32] but instead uses a subdivided frequency band [22]. The main frequency band (0–0.18 Hz) was decomposed into six subbands through wavelet packet decomposition. The main active channels were then extracted under each subband. This frequency range includes the metabolic activity of endothelial cells, myogenic activity, and respiratory activity except for neurotic activity. Different motion amplitudes can lead to the adjustment of metabolic frequency, respiratory frequency, and myogenic activity. Therefore, more subbands and the combination of multiple subbands are more conducive to characterize the differences between motion states. Moreover, to reduce the error caused by the subjects' different skull sizes and intersubject variability, the extraction of feature vectors focused on the key channels with the maximum or minimum values instead of focusing on fixed channels [22–24], and the channels were subdivided into more regions. Even though the number of features will increase a lot accordingly, the features will be simplified by statistical analysis when searching the common features among multistates in another dimension and different features between states in the current dimension (to be identified). Yin et al. [24] calculated the best accuracy rate through different combinations of hidden neurons and features, and Noori et al. [30] also recombined the feature vectors using a GA. In this study, the optimal feature vectors were identified and further simplified by the maximum training recognition rate attained by a combination of GA and SVM.

This study involved some deficiencies that should be improved. First, the purpose of this study was to propose a method of controlling walking-assistance equipment based on hemoglobin information to allow patients to walk independently. This study proposed a method to detect the motion intention of the subject, that is, it occurred before the actual motion. On the other hand, patients who do not have the ability to exercise generally have a certain degree of consciousness disorder and cannot normally imagine. Thus, this study designs an experimental paradigm of actual walking. However, all subjects involved in our experiments were healthy. Some studies have confirmed that the brain function of the elderly and stroke patients is different [15, 32–34]. Although this may be the case, the brain function subregions obtained by partitioning the brain function have a high degree of functional consistency [35]. Therefore, research based on healthy subjects provides a useful reference. For patients, the divided regions can be appropriately extended. In a subsequent study, experiments will be conducted using patients. Second, this study is mainly focused on classifying the motion intention with different gait states, but real-time BCI requires some consideration of the starting and end of the walking process. Thus, in future research, the major analysis should focus on this issue to enable dynamic classification, thus achieving real-time control of the walking-assistance equipment. Finally, although the recognition rate was not very high, it demonstrated the possibility of using the motion intention to identify walking speed (for different levels of step length) and step length (for different levels of walking speed). We think there are several possible reasons for this not high recognition rate. The first reason is that there is a coupling relationship between step length and walking speed: on the one hand, the brain function areas of walking speed adjustment and step length adjustment partially overlap; on the other hand, the step length is not constant when the subject is adjusting the walking speed. This is more complicated than identifying walking speed [36] or step length [37] alone. Another reason is the limit of the length of the road. In this walking experiment, because of our functional near-infrared spectroscopy equipment, the FORIE-3000 optical topography system is not portable. So the length of the road was approximately 4.4 m. This may cause a small sample size and cannot fully identify participants' walking intentions. In the future, using portable functional near-infrared spectroscopy equipment to collect the walking data at a long distance may improve the recognition rate. In this study, only the SVM algorithm was applied for the identification process. Hidden Markov models [32] and convolutional neural networks [38] could replace the SVM algorithm and may achieve better results.

## 5. Conclusion

By using the totalHb and the difference between oxyHb and deoxyHb, this study identified the spontaneous motion intention of step length and walking speed simultaneously, achieving recognition rates of walking speed (for different step lengths) and step length (for different walking speeds) of 71.21% and 71.21%, respectively. In this study, all the feature vectors were extracted before the actual movement, and the two-dimensional gait states were identified at the same time based on these feature vectors. For the feature selection, flexible channels were used to represent fixed regions, and ANOVA and GA were applied to identify the optimal feature vectors in 6 subbands. In conclusion, this study has laid the foundations for the control of walking-assistance equipment based on the motion intention of subjects to help patients to walk independently.

## Figures and Tables

**Table 1:** The classification of six gait states; a single state of one dimension contains one to three states of another dimension.

Classification condition	States to be identified	States of another dimension
Different levels of step length	LD	SP-LD, MP-LD
	MD	SP-MD, MP-MD, LP-MD
	HD	MP-HD
Different levels of walking speed	SP	SP-LD, SP-MD
	MP	MP-LD, MP-MD, MP-HD
	LP	LP-MD

SP-LD: the gait of small step with low speed; SP-MD: the gait of small step with midspeed; MP-LD: the gait of midstep with low speed; MP-MD: the gait of midstep with midspeed; MP-HD: the gait of midstep with high speed; LP-MD: the gait of large step with midspeed.

**Table 2:** Regional redistribution.

Region numbers	Corresponding channels	Region numbers	Corresponding channels
Re1	ch1, ch3, ch6	Re12	ch11, ch12, ch14
Re2	ch3, ch6, ch8	Re13	ch14, ch16, ch17
Re3	ch6, ch8, ch11	Re14	ch16, ch17, ch19
Re4	ch8, ch11, ch13	Re15	ch19, ch21, ch22
Re5	ch11, ch13, ch16	Re16	ch2, ch5, ch7
Re6	ch13, ch16, ch18	Re17	ch5, ch7, ch10
Re7	ch16, ch18, ch21	Re18	ch7, ch10, ch12
Re8	ch1, ch2, ch4	Re19	ch10, ch12, ch15
Re9	ch4, ch6, ch7	Re20	ch12, ch15, ch17
Re10	ch6, ch7, ch9	Re21	ch15, ch17, ch20
Re11	ch9, ch11, ch12	Re22	ch17, ch20, ch22

Region numbers: the brain region composed of the channel; Re1: region 1; Re2 to Re 22: the other twenty-one regions; ch1: channel 1; ch2 to ch22: the other twenty-one channels.

**Table 3:** The specific representation of serial number in Figures 6 and 7.

Serial	Combinations of feature vectors	Serial	Combinations of feature vectors
①	Re4: pd1(1) + pd2(1)	②	Re5: pd1(1) + pd2(1)
③	Re20: pd1(1) + pd2(1) + pd3(1)	④	Re21: pd4(1) + pd5(1) + pd6(1)
⑤	Re22: pd4(1) + pd5(1) + pd6(1)	⑥	Re5: pd4(1) + pd5(1) + pd6(1)
⑦	Re20: pd4(1) + pd5(1) + pd6(1)	⑧	Re21: pd4(1) + pd5(1) + pd6(1)
⑨	Re22: pd4(1) + pd5(1) + pd6(1)		
⑩	Re4: pd1(1) + pd2(1)	⑪	Re5: pd1(1) + pd2(1) + pd3(1)
⑫	Re6: pd1(1) + pd2(1)	⑬	Re11: pd1(1) + pd2(1)
⑭	Re15: pd1(1) + pd2(1)	⑮	Re20: pd1(1) + pd2(1) + pd3(1)
⑯	Re5: pd4(1) + pd5(1) + pd6(1)	⑰	Re6: pd4(1) + pd5(1) + pd6(1)
⑱	Re11: pd4(1) + pd5(1) + pd6(1)	⑲	Re15: pd4(1) + pd5(1) + pd6(1)
⑳	Re20: pd4(1) + pd5(1) + pd6(1)		

Re: the region; pd1: the frequency band in the 0-0.03 Hz range; pd2 to pd6: the other five frequency bands. The digital in the bracket represents the number of key channels in this region.

**Table 4:** The recognition rate of training set and testing set.

Classification dimension	States	Recognition rate of training set	Recognition rate of test set (correct number/total number)
Different levels of step length	LD	75%	71.21% (47/66)
	MD		
	HD		

Different levels of walking speed	SP	76.67%	71.21% (47/66)
	MP		
	LP		

LD: the gait of low speed; MD: the gait of midspeed; HD: the gait of high speed; SP: the gait of small step; MP: the gait of midstep; LP: the gait of large step.

## Data Availability

The .xlsx data used to support the findings of this study are included within the article.
